# Advances in Our Knowledge of Typhoid Fever

**Published:** 1899-06

**Authors:** 


### Advances in Our Knowledge of Typhoid Fever.

Since the sad experience of our troops at home and abroad last year with typhoid fever, medical interest in the disease has been, if possible, even more keen with regard to everything pertaining to it than ever before. The springtime nearly always witnesses a recrudescence of the disease in various parts of the country, owing to the fact that the melting snows and the spring freshets carry down with them into the water supplies of towns a certain amount of infective typhoid material that has been accumulating during the winter months. Typhoid is one of those diseases of which the practitioner is apt to think "there is nothing new under the sun," at least, nothing new that has a practical application, or is of value in the prophylaxis or treatment of the disease. A glance, we think, at Dr. Taylor's article on "Typhoid Fever" in *Progressive Medicine,* the new quarterly review of medical progress, edited by Professor Hare, is apt to disabuse one of such unprogressive notion.

With regard to prophylaxis of others during the treatment of a case of typhoid, these noteworthy recommendations from a French source are given: (1) Isolate patients suffering from typhoid fever, or at least do not permit them to be treated in a room or ward containing young people who have not previously had typhoid. The warning contains some wholesome advice too often neglected, and sometimes with sad results, because we are persuaded that typhoid is not an air-borne disease, and forget that contiguity favors infection because precautions will inevitably sometimes be neglected. (2) Nurses for typhoid cases should, if possible, be only such as have had typhoid themselves. In a family, the young people should be removed. (3) The floor of the sick room should be oiled, so as to be impermeable. Carpets and rugs should be removed, and the raising of dust should be avoided by frequent use of a cloth dampened with antiseptic solution. (4) The nurses should wear linen clothes, which they should remove when they leave the sick room, and in general they should be warned to be circumspect in their relations with others, and especially careful of the utmost details of antisepsis in the matter of the preparation of food and drink for themselves and others.

The review of the question of typhoid infection from oysters is full and conclusive. The possibility of typhoid infection through salads is made clearly apparent, manure being used in bleaching the plants and gardeners being careless in handling it and washing the plants in any sort of water, or sprinkling them with infected cistern water.

The strikingly practical features of this excellent review of the recent literature of typhoid, are the discussion of the question of typhoid without intestinal lesions, and of its corollary that intestinal lesions, even when existent, often play a very minor role in the disease. How important these questions are for the matter of treatment, is clear at once. All the so-called abortive methods of treatment, all the much-lauded systems or securing intestinal antisepsis, all the many drug formulæ and combinations that have been enthusiastically recommended for the treatment of typhoid, assume that the essence of the disease is the intestinal lesions. This is a notion that must disappear before scientific advance of our knowledge of the true nature of the disease.

